# Green-lipped (greenshell™) mussel (*Perna canaliculus*) extract supplementation in treatment of osteoarthritis: a systematic review

**DOI:** 10.1007/s10787-021-00801-2

**Published:** 2021-03-18

**Authors:** Maryam Abshirini, Jane Coad, Frances M. Wolber, Pamela von Hurst, Matthew R. Miller, Hong Sabrina Tian, Marlena C. Kruger

**Affiliations:** 1 School of Health Sciences, College of Health, Massey University, Private Bag 11222, Palmerston North, 4442 New Zealand; 2 School of Food and Advanced Technology, Massey University, Palmerston North, New Zealand; 3 Centre for Metabolic Health Research, Massey University, Palmerston North, New Zealand; 4 School of Food and Nutrition, Massey University, Auckland, New Zealand; 5 Cawthron Institute, Nelson, New Zealand; 6 Sanford Ltd., Auckland, New Zealand

**Keywords:** Greenshell mussel, Randomized-controlled trial, Osteoarthritis, Joint pain

## Abstract

**Objectives:**

Intervention studies using New Zealand green-lipped or greenshell™ mussel (GSM) (*Perna canaliculus*) extract in osteoarthritis (OA) patients have shown effective pain relief. This systematic review summarises the efficacy of GSM extracts in the treatment of OA.

**Methods:**

A literature search of the three databases EMBASE, MEDLINE, and Scopus was performed to identify relevant articles published up to March 2020. Inclusion criteria were clinical trials published in English measuring the effect of supplementation of whole or a lipid extract from GSM on pain and mobility outcomes in OA patients.

**Results:**

A total of nine clinical trials were included in systematic review, from which five studies were considered appropriate for inclusion in a forest plot. Pooled results showed that GSM extracts (lipid extract or whole powder) provide moderate and clinically significant treatment effects on a visual analogue scale (VAS) pain score (effect size: − 0.46; 95% CI − 0.82 to − 0.10; *p* = 0.01). The whole GSM extract improved gastrointestinal symptoms in OA patients taking anti-inflammatory medications. The GSM extract was considered to be generally well tolerated in most of the studies.

**Conclusion:**

The overall analysis showed that GSM provided moderate and clinically meaningful treatment effects on OA pain. However, the current evidence is limited by the number and quality of studies, and further larger and high-quality studies are needed to confirm the effectiveness and to identify the optimal GSM format. Nevertheless, it is worth considering using GSM extracts especially for patients seeking alternative pain relief treatments with fewer side effects compared to conventional treatment.

## Introduction

Osteoarthritis (OA) is the most common degenerative joint disease involving cartilage and surrounding tissues. It is a leading cause of disability worldwide, particularly among the elderly. A rise in OA incidence has correlated with growing populations of elderly and obese people (Cross et al. 2014; Litwic et al. 2013). The economic cost of OA is substantial; this includes not only medical-related expenses but also loss in work productivity (Altman 2010). The Osteoarthritis Research Society International (OARSI) guidelines recommend using analgesics and non-steroidal anti-inflammatory drugs (NSAIDs) to treat pain and inflammation (Bannuru et al. 2019). However, frequent and long-term use of these conventional therapies is often associated with adverse gastric events (Lamarque 2004), which has prompted OA patients to seek alternative and complementary medicines for managing their symptoms (Ramsey et al. 2001). Further studies for identifying effective and safer treatments are required. A range of nutritional supplements, including glucosamine and chondroitin sulphate have been widely used by OA patients. However, meta-analyses and reviews have revealed contradictory results. A recent meta-analysis concluded that the dietary supplements, glucosamine and chondroitin, were ineffective and provided only a small treatment effect on pain at medium- and long-term follow-up (Liu et al. 2018). In contrast, a second meta-analysis assessing 29 studies found oral supplementation with glucosamine or chondroitin sulphate significantly reduced pain in knee OA (Simental-Mendia et al. 2018).

*Perna canaliculus*, the green-lipped mussel, is endemic to New Zealand. It is grown for aquaculture only in New Zealand, where it is trademarked as the greenshell™ mussel (GSM). Various therapeutics such as Lyprinol^®^ are produced from GSM. When taken orally in whole powder or oil extract formats, GSM has been found to be beneficial for pain relief, reducing inflammation and ameliorating other debilitating symptoms associated with inflammatory diseases such as rheumatoid arthritis (RA) and OA without causing the adverse side effects of NSAIDs (Cho et al. 2003; Gibson and Gibson 1998). The underlying mechanisms explaining these effects are in the anti-inflammatory activity of bioactive lipids in mussels including eicosapentaenoic acid (EPA) and docosahexaenoic acid (DHA). These lipids mediate the inflammatory response by inhibiting both the cyclo-oxygenase (COX) and lipo-oxygenase (LOX) cascades of arachidonic acid (AA) metabolism, which results in a decrease in the synthesis of pro-inflammatory prostaglandins and leukotrienes (Halpern 2000; McPhee et al. 2007). Furthermore, GSM is reported to contain novel bioactive lipids such as pro-resolving lipid mediators which resolve inflammation through counter-regulating pro-inflammatory cytokines, clearing apoptotic neutrophils, and inducing wound healing and tissue regeneration (Wakimoto et al. 2011).

There have been only three systematic reviews evaluating results of clinical studies assessing the effects of GSM (whole or lipid extract) on joint symptoms of OA (Brien et al. 2008; Cobb and Ernst 2006; Ulbricht et al. 2009), and none was published in the last decade. These systematic reviews were conducted on only four or five randomized-controlled trials (RCTs). All three concluded that GSM extracts were beneficial as adjuvants to conventional therapies for arthritic conditions. However, these systematic reviews were limited by the number of studies and included studies with methodological deficiencies which prevented them from reaching comprehensive and reliable conclusions. Hence, a rigorous systematic review on this subject is needed to present updated and more conclusive evidence to evaluate the efficacy and safety of GSM supplements in clinical practice. In the present systematic review, we included a larger number of studies (*n* = 9) and larger sample size to provide more representative results. The aim of this analysis was to systematically review the existing clinical trials in the literature to evaluate the effectiveness of GSM supplementation in the treatment of OA symptoms.

## Materials and methods

### Data sources and searches

This systematic review was performed in accordance with the PRISMA statement (Moher et al. 2009). A systematic electronic search was conducted in the following databases from inception to March 2020: MEDLINE, Scopus, and EMBASE. A combination of relevant keywords including osteoarthritis, degenerative joint disorder, green-lipped mussel, *Perna canaliculus*, greenshell mussel, Seatone, Lyprinol, PCSO-524, and randomized clinical trial was applied to construct the search strategy. Initial screening of potentially relevant records was conducted based on title and abstract, and then, a final selection of included trials based on full-text evaluation was performed. In addition, the reference lists of included studies were reviewed for potential studies that were missed by the algorithm.

### Study selection and data extraction

Clinical trials published in English language were evaluated against the pre-defined criteria to be included in review. The eligibility criteria were: randomized-controlled or randomized non-controlled clinical trials; the GSM supplement was used as intervention in the form of lipid extract or freeze-dried whole powder (not in combination with other constituents with anti-inflammatory or anti-arthritic activity); the patients/participants were diagnosed with OA by radiography and outcomes of interest relevant to OA were assessed. There are no published reports identifying significant differences in treatment outcomes based on joint location, and a study of gene expression comparing OA versus normal cartilage found that the genes associated with OA were not correlated with the joint site (Ramos et al. 2014). Therefore, specifying the site of OA was not a requirement for inclusion in this review; this factor also enabled the inclusion of more available trials for assessment. A summary of each trial including the general study design; number of participants; dose and duration of intervention; concomitant medication; and outcome measures and adverse effects, is outlined in Table 1.

**Table 1:** General characteristic of the studies included in systematic review

Study, Year	Study design	Sample size (female/male) Mean age	Participant	Intervention and dosage	Control	Duration of study	Concomitant medications	OA outcome measures	Result	Jadad score
Clinical studies assessing the effect of GSM whole extract powder										
Gibson, 1980	Double-blind, randomized placebo-controlled	*I* = 16 *C* = 22 (37 F/1 M) 68.6 years	OA	Whole extract powder 1050 mg/day	Inactive fish capsule	3–6 months	NSAIDs	1. VAS 2. Morning stiffness 3. Functional index 4. Time taken to walk 50 feet 5. ROM of hip and knee joints 6. Patient's global assessment	The GSM extract improved VAS and stiffness No significant improvement in in ROM or grip strength was observed	1.5
Audeval, 1986	Randomized, double-blind, placebo-controlled trial	*I* = 27 *C* = 26 (37 F/16 M) 65 years	Knee OA	Six capsules of whole extract powder/day Dosage not Specified	Placebo	6 months	NSAIDs	1. ARA functional classification 2. Duration of morning Stiffness 3. Intensity of pain 4. Joint mobility 5. Distance from heel to cheek 6. Utilizing walking sticks 7. Patient's and physician's global assessment	The whole GSM extract improved ARA function, pain intensity and patients’ and physicians’ global assessment	2
Coulson, 2012	Non-blinded, Non-randomized pilot	*I* = 21 (13 F/8 M) 61.1 years	Knee OA	Whole extract powder (3000 mg/day)	None	8 weeks	NSAID, Paracetamol	Primary outcome: 1. WOMAC to assess pain, stiffness, and limitation of physical function 2. Lequesne algofunctional index to measure pain, walking distance, and activities of daily living Secondary outcome: 3. SF-12 to assess general quality of life 4. GSRS to assess gastrointestinal function	All outcomes were improved; however, SF-12 showed improvement for physical but not mental components of quality of life	1
Coulson, 2013	Randomized, non-blinded, Comparative controlled	*I* = 21 (16 F/5 M) 56.7 years *C* = 17 (12 F/5 M) 60 years	Knee OA	Whole extract powder (3000 mg/day)	Glucosamine sulphate	3 months	NSAIDs, paracetamol	Primary outcome: 1. Gut microbiota profile Secondary outcome: 2. WOMAC 3. Lequesne Algofunctional index 4. SF-12 GSRS	Both treatment group significantly improved all the OA outcome measures and GSRS score In mussel group *Bifidobacterium* tended to increase and *Enterococcus* and yeast species to decrease The *Clostridia species* tended to decrease from baseline to week 12 in both groups	3
Clinical studies assessing the effect of GSM lipid extract										
Gibson, 1998	Randomized, double-blind, comparative controlled for phase I, None-blind, non-controlled for phase II	*I* = 15 (10 F/5 M) 57.3 years *C* = 15 (12 F/3 M) 52.8 years	OA	Lipid extract 210 mg/day	Whole extract powder 1150 mg/day	3–6 months	NSAIDs	Assessment of pain and function: 1. VAS 2. Articular index 3. Morning stiffness 4. Grip strength 5. Night pain 6. Patient and physician's global assessments	Both GSM preparations improved all outcome measures, except for the VAS that improved only in GSM extract powder group	2.5
Cho, 2003	Non-randomized, non-blind, non-controlled trial	*I* = 54 (52 F/2 M) 61 years	Knee and/or hip OA	Four capsules of lipid extract/day Dosage not Specified	None	8 weeks	Use of NSAID was discontinued Use of rescue medication was not specified	Assessment of pain and function: 1. VAS, 2. Lequesne algofunctional index 3. Patient and physician's global assessment	Significantly improved all the outcomes	1
Lau, 2004	Randomized, double-blind, placebo-controlled trial	*I* = 40 (35 F/5 M) 62.1 years *C* = 40 (34 F/6 M) 62.9 years	Knee and/or hip OA	Lipid extract 4 capsules/day for 2 months and then 2 capsules/day for 4 months Dosage not Specified	Olive oil	6 months	Paracetamol, additional rescue medication	Assessment of pain and function: 1. VAS 2. Patient's and Physician's global assessment 3. COKS 4. CAIMS2-SF	Significantly improved all outcomes	3
Zawadzki, 2013	Randomized, blinded, comparative controlled in stage I, non-blinded in stage II	Phase I *I* = 25 (22 F/3 M) 65.58 years *C* = 25 (22 F/3 M) 66.72 years Phase II *I* = 22 (19 F/3 M) 67.23 years	Knee OA	Lipid extract 4 capsules 1200 mg/day	Fish oil 1200 mg/day	3–6 months	Paracetamol	Assessment of pain: 1. VAS (100 mm) Assessment of quality of life: 1. HAQ which cover eight categories of daily physical activities. These included dressing, arising, hygiene, walking, heating, eating, grip and daily activities 2. Health and Disease Condition	In phase I and II treatment with the lipid extract showed a significant improvement of VAS, HAQ all categories and health and disease condition Lipid extract did not show notable side effects while fish oil reported adverse side effect	4.5
Stebbings, 2017	Randomized, double-blind, placebo-controlled trial	*I* = 39 (22 F/17 M) 66.4 years *C* = 41 (22 F/19 M) 66.5 years	Knee and/or hip OA	Lipid extract enriched in NAE and long-chain omega-3 fatty acids 4 capsules 600 mg/day	Corn oil	3 months	NSAID, Analgesic	Assessment of pain and function: 1. WOMAC-pain scale 2. VAS (100 m) 3. Patient global assessment, 4. Total WOMAC score 5. WOMAC − 20 responder 6. Physician global assessment Assessment quality of life 7. HAQ 8. OAQol Change in analgesic use recorded in diary	No improvement on pain and quality of life (VAS, OAQol, WOMAC pain subscale, Physician and patient global assessment, HAQ, WOMAC − 20 responder, *p* > 0.05) Joint stiffness significantly improved WOMAC-stiffness subscale (*p* = 0.04) Significant difference in intake of paracetamol between the two groups (*p* = 0.001)	5

*I* intervention, *C* control, *ROM* range of movement, *HAQ* Health assessment questionnaire, *WOMAC* Western Ontario and McMasters OA Index, *OAQol* quality of life, *SF-12V2™* short from version 12 heath survey, *VAS* Visual analogue scale, *HAQ* Health assessment questionnaire, *GSRS* Gastrointestinal symptom rating scale, *COKS* Chinese version of the Oxford Knee Score, *ARA* American Rheumatism Association, *CAIMS2-SF* Chinese version of the Arthritis Impact Measurement Scale 2-short form, *CRP* C-reactive protein, *ESR* erythrocyte sedimentation rate, *NAE* N-acylethanloamine

### Risk of bias assessment

The five-point Jadad scale was applied to assess the quality of the included trials (Jadad et al. 1996). In this scale, the likelihood of bias is measured based on the description of randomization, blinding, and withdrawals from the trial on a scale of 0 (minimum) to 5 (maximum). Based on the total awarded score, studies with 0–2.5 scores were categorised as low quality and studies with 3–5 scores were defined as high quality (Table 1).

## Results

### Initial search and result

**Fig. 1:**
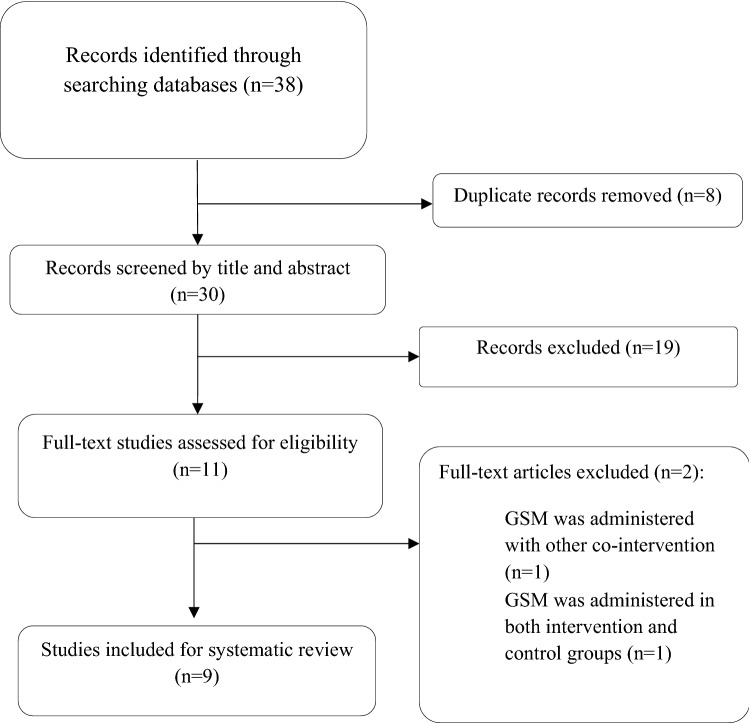
Flow diagram of study selection process

A flowchart of the search strategy and selection process is displayed in Fig. 1. A total of 38 citations were retrieved from the searched databases and after removal of duplicates, 30 publications were examined by title and abstract. Of these, 19 were discarded due to including non-OA patients, or not an RCT. The remaining 11 studies were selected for full-text reading and were also examined to ensure that the researchers had obtained ethical approval for the study. Of these 11 studies, two did not meet the inclusion criteria and were excluded: one study administrated GSM in both the intervention and comparator group (placebo) (Puente et al. 2014), and in the second study, GSM was administrated in combination with other bioactive ingredients (Qu et al. 2015). This resulted in a final total of nine studies to be included in this review.

### Study characteristics

The nine selected studies were published between 1980 and 2017. All studies involved both sexes with sample sizes ranging from 21 to 80 participants (Table 1). Across the studies, the average age of subjects ranged from 52.8 to 69.6 years. Four studies investigated only patients with knee OA (Coulson et al. 2012, 2013; Gibson and Gibson 1998; Lau et al. 2004). Three studies included patients with knee and/or hip OA (Cho et al. 2003; Stebbings et al. 2017; Zawadzki et al. 2013). Two studies did not specify the joint location (Gibson and Gibson 1998; Gibson et al. 1980).

The treatment and follow-up duration varied among studies from 8 weeks to 6 months. A variety of product preparations and different dosages were evaluated. GSM was used in the form of a whole extract powder in four studies, with doses ranging from 1050 to 3000 mg/day (Audeval and Bouchacourt 1986; Coulson et al. 2012, 2013; Gibson et al. 1980). Four studies used a lipid extract in doses ranging 210 and 1200 mg/day (Cho et al. 2003; Lau et al. 2004; Stebbings et al. 2017; Zawadzki et al. 2013). A single study compared a GSM lipid extract versus a whole GSM powder (Gibson and Gibson 1998). The included studies used olive oil (Lau et al. 2004), glucosamine sulphate (Coulson et al. 2013), corn oil (Stebbings et al. 2017), whole fish powder (Gibson et al. 1980), or fish oil (Zawadzki et al. 2013) as controls; one study used a non-specified placebo (Audeval and Bouchacourt 1986) and two studies lacked a comparator group (Cho et al. 2003; Coulson et al. 2012).

Seven of the nine included trials were parallel and randomized-controlled trials (Audeval and Bouchacourt 1986; Coulson et al. 2013; Gibson and Gibson 1998; Gibson et al. 1980; Lau et al. 2004; Stebbings et al. 2017; Zawadzki et al. 2013), and the remaining two were one-arm open-label trials (Cho et al. 2003; Coulson et al. 2012). Two studies included patients with both OA and RA, but their results were analysed and presented separately (Gibson and Gibson 1998; Gibson et al. 1980). Five of the nine studies were assessed to be of low quality (Jadad score of 1–2.5), and four studies were assessed to be of high quality (Jadad score of 3–5). According to the study quality assessment using the Jadad scale, only one study fulfilled all the criteria required to reach a score of 5 (Stebbings et al. 2017).

A range of outcomes were measured using standardised assessments, the most prevalent of which were the self-reported Visual Analogue Scale (VAS) for pain and the Western Ontario and McMasters OA Index (WOMAC) for OA outcome measurements (Coulson et al. 2012, 2013; Stebbings et al. 2017). Other standardised tests included the patient's and/or physician's global assessment, the Lequesne Algofunctional Index for OA severity, quality of life assessed by Health assessment questionnaire (HAQ), the short-form version 12 health survey (FS-12), and the Osteoarthritis Quality of Life (OAQol) assessment. Individualised or customised assessments were also performed including duration of morning stiffness, time taken to walk a fixed distance, grip strength, and assessment of gastrointestinal function or microbiota.

### Data synthesis and analysis

To enhance a visual inspection of favourable GSM extract results over placebo or a comparator, data from controlled trials that included the VAS pain scale as a primary outcome were pooled using a random-effects model. The mean difference (MD) of VAS between control and intervention groups was applied to calculate the overall effect size of the intervention. The effect size was expressed as the standardised mean difference (SMD) and 95% confidence interval (CI) from the random-effects model. We applied the following formula to calculate the standard deviation (SD) of the MD for studies that did not report this parameter: SD^2^ = [(SD baseline^2^ + SD final^2^) − (2 × 0.8 × SD baseline × SD final)]. The heterogeneity across studies were evaluated by the *I*^2^ statistic. We considered the treatment effect small if the effect size was < 0.3, moderate if the effect size was between 0.3 and 0.8, and large if the effect size was > 0.8. To interpret whether the effect size was clinically important, a threshold of 0.37 standardised units was considered as the minimum clinically important difference (MCID); this is based on the previous reviews (da Costa et al. 2017; Liu et al. 2018). The analyses were performed using STATA version 14 (StataCorp, College Station, TX, USA). A *p* value < 0.05 was considered statistically significant.

### Forest plot analysis

**Fig. 2:**
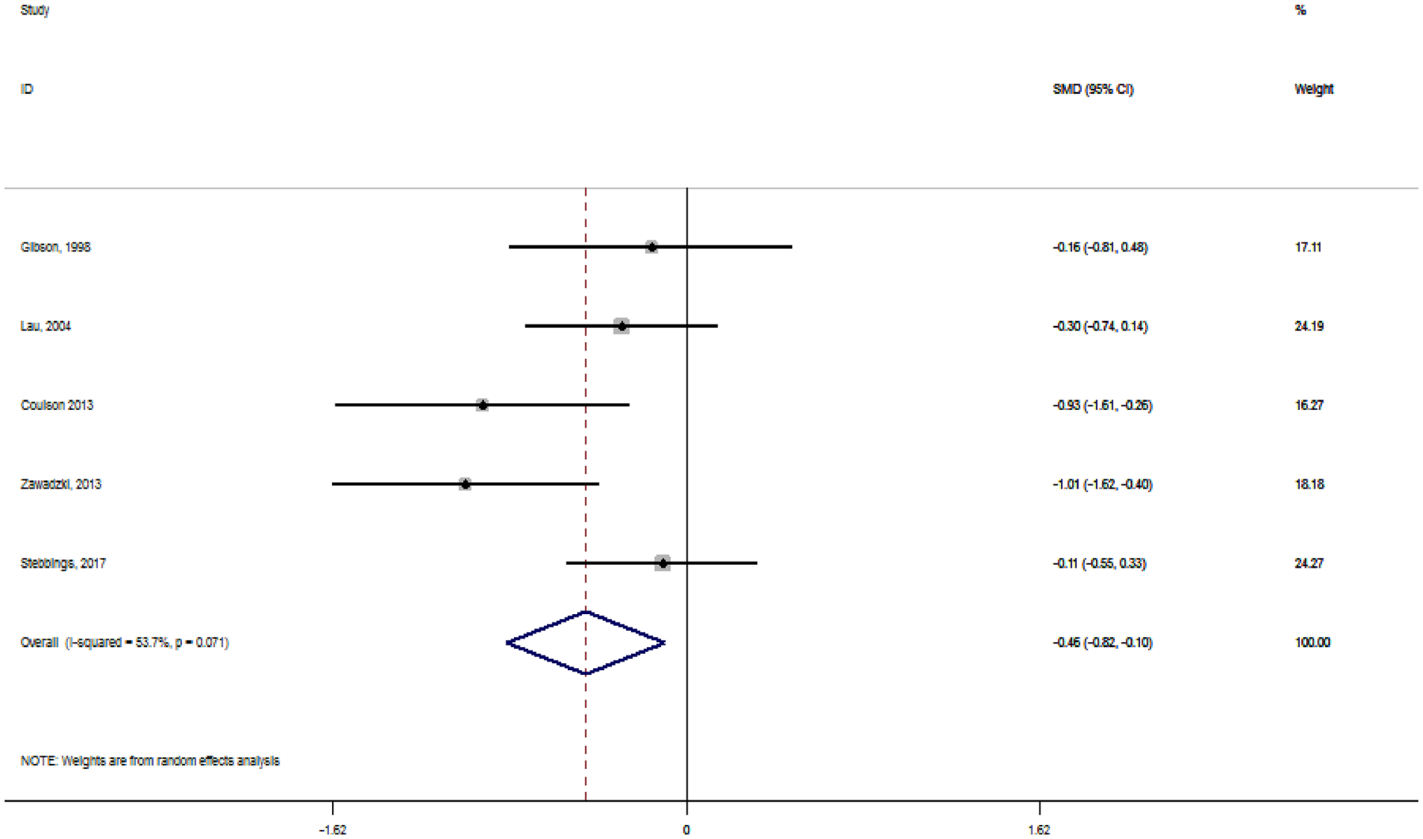
Forest plot displaying standard mean difference (SMD) and 95% confidence interval (CI) measuring the impact of GSM supplementation on VAS pain score. The black dots estimate the study result and give a representation of the size of the study. A horizontal line representing the 95% confidence intervals of the study result, with each end of the line representing the boundaries of the confidence interval. The diamond represents the effect size and confidence intervals that combines all the individual studies together

From nine included studies, five were considered appropriate for inclusion into a forest plot analysis. Four studies were excluded, because they did not assess the VAS as outcome or were non-controlled clinical trials. The pooled analysis identified moderate and clinically important effects of GSM supplementation in reducing the VAS pain score (SMD: − 0.46, 95% CI − 0.82 to − 0.10, *p* = 0.01), as shown in Fig. 2. However, this result was collated from a limited number of studies (*n* = 5) with participant numbers ranging from 30 to 80 (278 cumulative participants). There was substantial heterogenicity among the included studies (*I*^2^ = 53.7%, *p* = 0.07). We were unable to explore potential sources of heterogeneity or the influence of different factors on the treatment effect due to the limited number of studies available for assessment.

## Discussion

We performed a comprehensive systematic review on the efficacy of both types of GSM preparations (whole stabilised powder and lipid extract) in treating knee and hip OA symptoms, including nine clinical trials and 452 participants. The OA signs, symptoms, and physical function of each patient were assessed by the research groups using various parameters. VAS, the most prevalent measure for pain assessment, revealed a significant reduction in pain favouring the intervention group. Improvements in stiffness, physical function, and physical aspects of quality of life due to the GSM intervention were also observed in most of the studies. However, the mental component of quality of life did not change meaningfully. Overall, our review demonstrates that GSM products are moderately effective in reducing the VAS pain score in OA patients. With the exception of some minor gastrointestinal adverse side effects reported by one research group (Coulson et al. 2012, 2013), both lipid and powdered whole extract products were generally well tolerated and safe to use.

The earliest studies using freeze-dried GSM powder reported a trend for improvement in pain and stiffness compared to a placebo (Audeval and Bouchacourt 1986; Gibson et al. 1980). However, a notable weakness of these earlier studies is a lack of stability of the mussel products, which strongly affected their anti-inflammatory potency. Moreover, these studies did not provide information on the dose of NSAID medications used by the participants, and generally were of low quality and lacking in rigorous methodology (Whitehouse et al. 1997). There was only one RCT identified in the literature which compared the efficacy of a lipid extract and stabilised whole GSM powder in OA patients (Gibson and Gibson 1998). In this randomized, double-blinded, controlled clinical trial with a parallel design, patients received the comparatively low dose of 210 mg/day lipid extract or 1150 mg/day stabilised GSM powder for 3 months, after which all patients received the lipid extract for a further 3 months. The evaluation by the patients and physicians revealed a significant improvement in stiffness and function in both intervention groups as reported by 70% of patients. The results of this study showed no difference between the degree of efficacy and the required time to show efficacy in lipid extract versus stabilised powder groups (Gibson and Gibson 1998). However, a significant improvement in the VAS pain scale was observed only in the whole GSM powder group as 70% of patients benefited from it. This intriguing finding suggests that non-lipid components contributed to the health benefits, given that GSM has been shown to contain < 2% lipids by wet weight (Miller et al. 2014), and approximately 8% lipids by dry weight (Siriarchavatana et al. 2019); thus, 1150 mg GSM powder would contain < 100 mg lipid, which is less than half of the dose given to the lipid-group participants. Further studies are clearly needed to determine the relative contributions of GSM lipids, proteins, and other components to its beneficial effects in reducing OA symptoms.

In two other studies in which a high dose of GSM whole extract powder (3000 mg/day) was administered to patients with knee OA, subjects in the intervention group exhibited improvements in OA outcome measures (Coulson et al. 2012, 2013). The first study was an open-label, single group pilot study, and the subsequent one was a non-blinded randomized, comparator-controlled trial. It is worth noting that the first study was limited by a small sample size (*n* = 21) and short duration (8 weeks) (Coulson et al. 2012). The first study showed that the WOMAC subscales and Lequesne algo functional index as outcome measures for pain and function were improved; however, the general quality of life assessed through SF-12 showed improvement only in terms of physical components and not mental components. The follow-up study included a larger sample size (*n* = 40) and compared 3000 mg/day of GSM whole extract powder with 3000 mg/day of glucosamine sulphate for a longer period of 12 weeks (Coulson et al. 2013). In this study, both groups reported significant improvements in the same outcomes as the first study, but no significant differences between the two interventions were observed. Interestingly, both studies showed that GSM whole extract powder significantly attenuated the detrimental gastrointestinal aspects assessed by GSRS scores, which are linked to the long-term use of NSAID medications. In addition to measuring gastrointestinal function, the second study assessed the gut microbiota profile. Both whole GSM extract and glucosamine sulphate supplementation resulted in slight changes in the microbiota profiles from baseline, with the most notable being a decrease in the *Clostridia* species. These bacteria can induce a T-cell-driven gastrointestinal inflammatory response (Stepankova et al. 2007), and the decrease in *Clostridia* occurred concurrently with both a decrease in inflammation and improvement in gastrointestinal and joint symptoms. These results further suggest that alteration in gut microbiota profiles should be regarded as a critical factor in determining the therapeutic efficacy of nutraceutical interventions such as GSM and glucosamine for OA treatment (Coulson et al. 2013). Additional studies are warranted to confirm the interaction of GSM bioactive compounds with intestinal microbiota.

A few studies have assessed the efficacy of GSM lipid extracts in treating OA. The Lau study (Lau et al. 2004) involving knee OA patients identified a positive benefit of GSM lipid extract versus olive oil control over 6 months. In this study, all the arthritis assessment parameters, including VAS, patient’s and physician’s global assessment of arthritis, a validated Chinese version of the Oxford Knee Score (COKS), and a validated Chinese version of the Arthritis Impact Measurement Scale 2-short form (CAIMS2-SF) that assessed physical function and psychological status, were significantly improved in the lipid extract group. The greatest improvements from week 4 associated with GSM lipid were in the VAS pain perception and patients’ global assessment of arthritis. Consistent with this study, an RCT by Zawadzki et al. (2013) compared equal doses (1200 mg/day) of a GSM extract containing 5.2% EPA and 3.4% DHA versus fish oil containing a standardised amount of 18% EPA and 12% DHA in patients with knee and/or hip OA. Significant and positive effects on VAS pain scale quality of life and overall health condition were reported in the GSM group compared to the fish oil group. There was an 89% decrease in the VAS pain score and 91% of patients reported an improvement in quality of life. No apparent adverse effects were observed in lipid extract group, while patients treated with fish oil showed a less meaningful reduction in VAS pain score, and an insignificant quality of life score accompanied by some adverse effects. The researchers suggested that larger doses and a longer duration were required for fish oil to exhibit efficacy. Overall, this study concluded that the GSM lipid extract was a safer alternative and resulted in faster and more notable treatment effects with smaller dosages compared with fish oil. It is of interest to note that the GSM resulted in significantly better health outcomes in this study, even though the fish oil intervention contained threefold higher amounts of EPA and DHA, suggesting that other components in GSM provided the bioactivity. This result is consistent with evidence that the GSM lipid fraction contains various lipid bioactives and unique fatty acids such as tetraenoic acids with great anti-inflammatory activity (Sinclair et al. 2000). These fatty acids have been demonstrated to compete much more efficiently than AA for the COX and LOX pathways (Treschow et al. 2007). Furthermore, furan fatty acids are unstable anti-inflammatory and antioxidant components detected in GSM in minor amounts. In a study by Wakimoto and co-authors that employed the semisynthetic route from the furan dicarboxylic acid (the shark metabolite of furan fatty acids), furan fatty acid ethyl esters showed more potent anti-inflammatory effects than EPA in a rat model of adjuvant-induced arthritis (Wakimoto et al. 2011).

In the most recent RCT reported by Stebbings (Stebbings et al. 2017), 80 patients with moderate-to-severe OA were randomly provided with a novel GSM lipid extract enriched in N-acylethanloamine (NAE) and long-chain omega-3 fatty acids (600 mg/day) versus corn oil as placebo over 12 weeks. In contrast to previous findings, the results showed no statistically significant difference between the intervention and placebo groups in pain or quality of life outcomes measured by various assessments including VAS, WOMAC-pain subscale, patient’s and physician’s global assessment, OAQol, and HAQ. However, joint stiffness measured by WOMAC-stiffness subscale improved in the lipid extract group compared to the placebo. In addition, patients in the lipid extract arm reduced their intake of paracetamol in comparison to the placebo group and this effect continued for 3 weeks after the intervention ceased at week 12. The researchers suggested that significant pain improvement was not detected due to the severity of disease in this cohort, as half of the patients had moderate-to-severe OA. This suggests that GSM may have greater effect on stiffness rather than pain perception in severe OA. Alternatively, the GSM product used in this study may have lacked specific bioactive lipids; in previous studies that obtained positive results with high doses of freeze-dried powdered products (Coulson et al. 2013) or lipid extracts (Zawadzki et al. 2013), the test products were likely to have contained different profiles of lipids compared to the product used by the Stebbings group. It would be interesting for future RCTs to assess pain and stiffness improvements in patients with differing stages of OA severity, and to identify the precise components that provide the bioactivity.

One limitation in all the studies included in this review was the uneven distribution of women to men, in favour of the former. All recruits were middle-aged or elderly, and all studies included participants of both sexes, but females predominated (nearly 80%). Because of this, the findings may not extrapolate to the male population. A second limitation was the absence of data on the menopausal status or use of hormone therapy by female participants. After reaching menopause, women have a higher risk and present more advanced stages of disease and more debilitating OA pain compared to age-matched males (Hame and Alexander 2013; Tanamas et al. 2011). There is also evidence, suggesting that oestrogen therapy reduces risks of joint symptoms (Chlebowski et al. 2013). Therefore, future studies should specifically include the menopausal and hormonal status of female participants. A third limitation is duration of study: half of the included studies in this review were carried out for ≤ 3 months. It cannot be determined whether GSM supplements would have retained their efficacy for a longer period, or conversely whether additional health benefits would have been observed. Finally, it is worth noting that the number of studies available for this review was limited, which emphasises the need for further RCTs to establish optimal treatments and doses. These studies should consider controlling for gender differences, monitor adverse effects, measure changes in gut microbiota, and employ standardised measurements for assessing OA symptoms.

## Conclusion

This review expands on previous reports of GSM supplementation as a treatment for OA, as it has assessed a larger number of studies. Although most of the included studies were funded by pharmaceutical companies, the companies did not play roles in data analysis or interpretation, reducing the likelihood of bias. The forest plot for VAS pain outcome in this review was able to clearly demonstrate moderate efficacy of GSM over placebos or other interventions. From the evidence currently available, we conclude that the use of GSM for patients with OA provides benefit for pain relief and does not cause significant negative side effects. Even in the absence of further studies to provide additional data, GSM extracts can be recommended to OA patients who seek alternative options for pain improvement with fewer gastrointestinal side effects.
